# Professional burnout syndrome in European primary care doctors: A joint call for system-level action between the European Psychiatric Association (EPA) and the World Organization of National Colleges, Academies and Academic Associations of General Practitioners/Family Physicians-Europe Region (WONCA Europe)

**DOI:** 10.1192/j.eurpsy.2026.10152

**Published:** 2026-01-26

**Authors:** Ferdinando Petrazzuoli, Martina Rojnic Kuzman, Julian Beezhold, Shlomo Vinker, Thomas Frese, Andrea Fiorillo

**Affiliations:** 1 World Organization of National Colleges, Academies and Academic Associations of General Practitioners/Family Physicians-Europe Region (WONCA Europe); 2 Department of Clinical Sciences, Centre for Primary Health Care Research, Lund University, Malmö, Sweden; 3 WONCA Working Party on Mental Health, Italy; 4 European Psychiatric Association, Croatia; 5 Department of Psychiatry, Zagreb University Hospital Centre, Kispaticeva, Zagreb, Croatia; 6 Zagreb School of Medicine, Salata, Zagreb, Croatia; 7 Great Yarmouth Acute Service, Northgate Hospital/Norfolk & Suffolk NHS Foundation Trust, Great Yarmouth, United Kingdom; 8 Norwich Medical School, University of East Anglia, Norwich, United Kingdom; 9 Leumit Research Institute, Leumit Health Services, Tel Aviv, Israel; 10 Institute of General Practice and Family Medicine, Martin-Luther-University Halle-Wittenberg, Halle, Germany; 11 Department of Psychiatry, University of Campania “L. Vanvitelli”, Naples, Italy

**Keywords:** Europe, interventions, prevalence, primary care, professional burnout syndrome, physician well-being, risk factors

## Abstract

Professional burnout syndrome represents a significant occupational hazard within European primary care physicians, impacting their well-being, quality of care, and the sustainability of healthcare systems. This joint European Psychiatric Association (EPA) and the World Organization of National Colleges, Academies and Academic Associations of General Practitioners/Family Physicians- Europe Region (WONCA Europe) viewpoint focuses specifically on primary care physicians, contrasts their risk profile with other specialties, and outlines actionable, system-level recommendations for policymakers, provider organizations, and professional associations. Evidence indicates a wide range in professional burnout syndrome prevalence, influenced by assessment methodologies and specific national contexts. The syndrome manifests through emotional exhaustion, depersonalization, and reduced personal accomplishment, often accompanied by secondary psychological and physical symptoms. A multitude of interacting risk factors at the individual, interpersonal, and organizational levels contribute to its development. Effective mitigation strategies necessitate a multi-pronged approach encompassing individual coping mechanisms and systemic organizational changes aimed at alleviating workload, enhancing autonomy, and fostering supportive work environments.

## Introduction

Professional burnout syndrome among health care professionals is a widespread concern in health care both before and after the COVID-19 pandemic.

Although professional burnout syndrome is included in the 11th Revision of the International Classification of Diseases (ICD-11) as an occupational phenomenon and not classified as a medical condition, the consequences on the people affected are not to be underestimated [1]. According to the ICD-11 classification, three symptoms define the syndrome: (i) feelings of energy depletion or exhaustion; (ii) increased mental distance from one’s job or feelings of negativism or cynicism towards one’s job; and (iii) a sense of ineffectiveness and lack of accomplishment. A recent systematic review highlighted the complexity of the management and prevention of work-related stress, which requires a multicomponent and multilevel approach. Furthermore, this may be even more pronounced among women, who often experience higher work-family conflict, which in turn may contribute to the gender gap in sick leave [2–4].

Professional burnout syndrome, as defined by Maslach and Jackson [5], is a psychological syndrome characterized by three distinct dimensions: emotional exhaustion (feelings of being overextended and depleted of emotional resources), depersonalization (a cynical and detached response towards patients) and reduced personal accomplishment (feelings of incompetence and a lack of achievement in one’s work). The ramifications of professional burnout syndrome extend beyond individual physician’s well-being, impacting patient care quality, physician retention, and the overall efficiency of healthcare delivery.

Primary care represents the cornerstone of healthcare systems across Europe, providing accessible and comprehensive care to diverse populations. However, the demanding nature of primary care practices places significant strain on physicians, rendering them vulnerable to professional burnout syndrome. Although professional burnout syndrome was previously present, the COVID-19 pandemic has shown that the primary care workforce is at high risk of professional burnout syndrome [6, 7].

The magnitude of this problem has also been recently acknowledged by the World Health Organization (WHO) and the Organization for Economic Co-operation and Development (OECD). In a recent WHO Europe meeting in Bucharest on health and care workforce, it was noted that “this long-standing challenge which the COVID-19 pandemic has exacerbated, has also highlighted the need to protect the mental and physical health and well-being of workers, many of whom continue to experience stress, professional burnout syndrome and violence, with some leaving their jobs” [8].

In a survey carried out in 2022, data from 10 OECD countries show that, on average, almost 40% of primary care doctors under age 55 report feelings of burnout, and over 10% intend to stop seeing patients in the near future. Less than one in five primary care physicians across the 10 OECD countries reported high satisfaction with their work-life balance in 2022 [9].

Development of this viewpoint: This joint statement was drafted by a working group of the European Psychiatric Association (EPA) and WONCA Europe, informed by recent international guidance and surveys, and refined through internal consultations across both organizations. This viewpoint focuses specifically on primary care physicians, contrasts their risk profile with other specialties, and outlines actionable, system-level recommendations for policymakers, provider organizations, and professional associations.

## Prevalence of professional burnout syndrome in European Primary Care

The reported prevalence of professional burnout syndrome among European primary care physicians exhibits considerable heterogeneity, attributable to variations in assessment tools, diagnostic criteria, and the specific populations studied. A seminal cross-sectional study encompassing 12 European countries [7] revealed that a substantial proportion of family doctors experienced high levels of emotional exhaustion (43%), depersonalization (35%), and low personal accomplishment (32%), with 12% of them meeting the criteria for high professional burnout syndrome across all three dimensions.

A more recent systematic review by Wahlin et al. [10] analyzing data from 41 European countries, further highlighted this variability, reporting a professional burnout syndrome prevalence ranging from 2.5% to 72.0%. When employing a strict tridimensional definition of professional burnout syndrome, the pooled prevalence rate was estimated at 7.7%. However, broader definitions – incorporating elevated scores in any one or two dimensions – yielded significantly higher prevalence rates, highlighting the sensitivity of prevalence estimates to methodological approaches. Notably, specific events, such as the COVID-19 pandemic, have been shown to exacerbate professional burnout syndrome levels. A study in Catalonia, Spain, documented a dramatic surge in the levels of professional burnout syndrome across all three dimensions among primary care physicians, increasing from 10% pre-pandemic to 50% during the pandemic’s peak [11]. These findings show the dynamic nature of professional burnout syndrome and its susceptibility to acute stressors within the healthcare environment.

## Symptoms of professional burnout syndrome in Primary Care Physicians

Professional burnout syndrome in primary care physicians manifests through a constellation of interconnected symptoms that span emotional, interpersonal, and personal domains [2].

- *Emotional Exhaustion*: This core dimension is characterized by feelings of being emotionally drained and fatigued due to the cumulative demands of patient care and the healthcare system. Physicians may report feeling overwhelmed, irritable, and lacking the energy to engage effectively with patients and colleagues.
- *Depersonalization:* Also referred to as cynicism, depersonalization involves the development of negative, detached, and often dehumanizing attitudes towards patients. Physicians experiencing depersonalization may view patients as mere cases or burdens, exhibiting a lack of empathy and emotional distance in their interactions.
- *Reduced Personal Accomplishment:* This dimension reflects feelings of inadequacy and a decline in one’s sense of professional effectiveness and achievement. Physicians may perceive their efforts as futile, question their competence, and experience a reduced sense of satisfaction from their work.

Beyond these core dimensions, professional burnout syndrome can also manifest with secondary symptoms, such as increased irritability, anxiety, sleep disturbances, difficulties in concentrating, decreased motivation, and various physical complaints, including headaches and gastrointestinal problems. The presence and severity of these symptoms can significantly impact physicians’ overall well-being and their ability to provide high-quality patient care.

## Risk Factors for professional burnout syndrome in European Primary Care

The development of professional burnout syndrome in European primary care physicians is a complex interplay of individual vulnerabilities and a multitude of occupational stressors.

- *Workload and Time Pressure*: The demanding nature of primary care, characterized by high patient volumes, short consultation times, and increasing administrative responsibilities, places immense pressure on physicians, contributing significantly to emotional exhaustion.
- *Administrative Burden:* The escalating burden of paperwork, documentation requirements, electronic health record management, and other administrative tasks detracts from direct patient care and has been consistently identified as a major contributor to professional burnout syndrome [12].
- *Lack of Control:* Limited autonomy over work schedules, clinical decision-making processes, and organizational policies can foster feelings of helplessness and contribute to professional burnout syndrome.
- *Work Environment:* A chaotic or poorly organized work environment, characterized by inadequate staffing, lack of resources, and poor communication, can exacerbate stress and increase the risk of professional burnout syndrome.
- *Emotional Demands:* The inherent emotional intensity of primary care, involving exposure to patient suffering, managing complex psychosocial issues, and delivering difficult news, can be emotionally draining and contribute to emotional exhaustion and depersonalization.
- *Work-Life Imbalance*: Difficulty in maintaining a healthy boundary between professional and personal life, often due to long working hours and the pervasive nature of healthcare demands, significantly increases the risk of professional burnout syndrome.
- *Demographic Factors:* Some studies suggest that younger physicians and female physicians report higher levels of specific professional burnout syndrome dimensions, although findings remain inconsistent across different European contexts.
- *Job Dissatisfaction:* Low levels of job satisfaction, often stemming from the stressors above, are strongly associated with higher rates of professional burnout syndrome across all dimensions, including feelings of not having enough support and being disconnected from other specialties while at the same time being at the front line for patients.

## Interventions to mitigate professional burnout syndrome in European Primary Care

Addressing the pervasive issue of professional burnout syndrome in European primary care necessitates a comprehensive and multi-level approach targeting both individual physicians and the organizational systems within which they operate. Preventing and detecting professional burnout syndrome is primarily a systems issue requiring proactive leadership. Good organizational leadership requires the implementation of systematic prevention measures alongside early-warning detection.

### Individual-focused interventions

- *Stress Management Training:* Equipping physicians with evidence-based stress management techniques, such as mindfulness-based stress reduction (MBSR) and cognitive behavioral therapy (CBT), can enhance their coping mechanisms and resilience.
- *Time Management and Prioritization Skills:* Training in effective time management strategies and prioritization techniques can help physicians manage their workload more efficiently and reduce feelings of being overwhelmed.
- *Promoting Social Support:* Encouraging peer support groups, mentorship programs, and access to counselling services can provide physicians with crucial emotional support and a sense of community.
- *Enhancing Self-Care Practices:* Promoting healthy lifestyle choices, including regular exercise, adequate sleep, and balanced nutrition, alongside encouraging dedicated time for personal interests and relaxation, can bolster individual well-being and resilience.

### Organizational-level interventions

- *Workload Reduction and Optimization:* Implementing strategies to manage patient volumes, increase the number of family physicians and their national even distribution, optimize scheduling practices, and increase administrative support can alleviate time pressure and reduce workload.
- *Streamlining Administrative Processes:* Reducing unnecessary administrative burdens through efficient electronic health record systems, standardized documentation protocols, and dedicated administrative staff can free up physician time for direct patient care [12].
- *Enhancing Autonomy and Control:* Providing physicians with greater input into their schedules, clinical guidelines, and organizational decision-making processes can foster a sense of ownership and reduce feelings of powerlessness.
- *Improving the Work Environment:* Cultivating a supportive and collaborative work culture, promoting open communication, fostering teamwork, and ensuring adequate resources can create a more positive and less stressful work environment [13].
- *Leadership Development:* Training healthcare leaders to recognize the signs of professional burnout syndrome, promote physician well-being, and implement supportive policies is crucial for creating a professional burnout syndrome-aware organizational culture [14].
- *Implementing Team-Based Care Models*: Adopting team-based care approaches, involving nurses, physician assistants, and other allied health professionals, can distribute workload and provide peer support, potentially mitigating professional burnout syndrome among physicians. Training in multisectoral or multidisciplinary approach together with other specialties in treatment of complex disorders that require several types of professionals, such as mental health conditions, and including social workers and psychologists.
- *Wellness Programs:* Offering institutional wellness programs that provide access to mental health resources, workshops on stress reduction, and facilities for physical activity can proactively support physician well-being.
- *Flexible Work Arrangements:* Exploring options for part-time work, job sharing, and flexible scheduling can help physicians to achieve a better work-life balance [14].
- *Interventions aimed at reducing gender gaps:* Evaluating the gender differences within each professional burnout syndrome driver and developing sustainable strategies to reduce disparities, support career and family planning [4, 15].

## Recommendations for policymakers, provider organizations, and professional bodies

### A. Policy (policy makers)

- What: Reduce low-value administrative load; align payment with team-based care and continuity; and fund primary care staffing.
- How: Set national targets to reduce administrative time (e.g., simplify sick-leave certifications; streamline repeat prescribing); fund medical assistants/care coordinators; and support interoperable EHRs.
- Expected outcomes: Reduced after-hours EHR time, improved continuity and access, lower turnover.

### B. Provider organizations (primary care networks, clinics)

What: Implement team-based care and schedule control; measure professional burnout syndrome like a safety metric.

How: Minimum standards for appointment length; protected, rostered administrative blocks; standardized triage; care-navigation roles; and regular burnout pulse checks with feedback-to-action.

Expected outcomes: Lower emotional exhaustion, greater job control, better patient experience.

### C. Professional organizations (WONCA Europe, EPA)

WONCA Europe will: (i) operationalize the Overdiagnosis/Overtreatment (“too much medicine”) position into CPD modules; (ii) issue a practical “Choosing Wisely in Primary Care” starter set for Europe; and (iii) advocate EU/Member State funding for team-based primary care.

EPA will: (i) co-develop brief, scalable manager training for clinic leads; (ii) curate evidence-based peer-support/reflective practice formats for primary care; (iii) publish joint guidance on safe pathways for primary care doctors to access mental-health support without stigma; (iv) co-develop recommendations for integration/ strengthening cooperations of primary health care into the operating system for mental health care [16].

Joint: advocate adoption of WHO mental-health-at-work guidance in primary care and co-monitor progress annually.

### D. Education and culture

What: Embed “too much medicine” (“Too much medicine” refers to avoidable cascades of testing and treatment that do not improve outcomes and may cause harm; addressing overuse through education and practice redesign is a lever to reduce workload and moral injury in primary care.) and cognitive ergonomics into undergraduate, postgraduate, CPD training, and innovative models of organization of primary health care, which take into account mental health care as suggested by WHO [16].

How: Case-based modules; audit/feedback on overuse; incorporate living guidelines addressing overuse and underuse.

Expected outcomes: Reduced low-value care and moral injury; increased professional fulfillment and reduced burden in everyday work arising from better cooperation with other professionals.
